# Epigenetic silencing of the MUPCDH gene as a possible prognostic biomarker for cyst growth in ADPKD

**DOI:** 10.1038/srep15238

**Published:** 2015-10-14

**Authors:** Yu Mi Woo, Yubin Shin, Jung-Ah Hwang, Young-Hwan Hwang, Sunyoung Lee, Eun Young Park, Hyun Kyung Kong, Hayne Cho Park, Yeon-Su Lee, Jong Hoon Park

**Affiliations:** 1 Department of Biological Science, Sookmyung Women’s University, Seoul, 140-742, Korea; 2 Branch of Cancer Genomics, Research Institute, National Cancer Center, Goyang-si, Gyeonggi-do, Korea; 3 Department of Internal Medicine, Eulji General Hospital, Seoul, 139-892, Korea; 4 Division of Nephrology, Armed Forces Capital Hospital, Seongnam, Korea

## Abstract

Although autosomal dominant polycystic kidney disease (ADPKD) is a common genetic disease, and is characterized by the formation of multiple fluid-filled cysts, which results in renal failure, early diagnosis and treatment of ADPKD have yet to be defined. Herein, we observed that the promoter region of the gene encoding mucin-like protocadherin (*MUPCDH*) was hypermethylated in the renal tissue of patients with ADPKD compared to non-ADPKD controls. Inversely, *MUPCDH* was significantly repressed in ADPKD, especially in cyst-lining cells. Our results indicate that aberrant methylation of *MUPCDH* promoter CpG islands may be negatively correlated with reduced expression level of *MUPCDH* and that this contributes to abnormal cell proliferation in ADPKD. It suggests that methylation status of *MUPCDH* promoter can be used as a novel epigenetic biomarker and a therapeutic target in ADPKD.

Autosomal dominant polycystic kidney disease (ADPKD) is the most common inherited systemic disorder, with an incidence of 1 in 500–1,000 individuals^1^. ADPKD is characterized by the development of numerous fluid-filled cysts in both kidneys, and this eventually leads to end-stage renal failure^2^,^3^. In particular, increased proliferation of renal tubular epithelial cells is considered as a major factor driving cyst formation and expansion in ADPKD^4^,^5^,^6^. ADPKD is caused by mutations in the *PKD1* and *PKD2* genes, which encode polycystin−1 and −2, respectively^7^. However, despite major causative genes being identified, the mechanism of cyst formation remains unclear, and there are no effective therapeutic targets for ADPKD. Currently, the diagnosis of ADPKD is routinely based on the detection of kidney cysts via ultrasound imaging, which has limited sensitivity in patients with a mild disease phenotype, such as children, young adults, and those with *PKD2* mutations, even if they have a family history of ADPKD^8^. Moreover, as the glomerular filtration rate (GFR) remains stable for many decades in the early disease stage, even with the formation of multiple cysts^9^, the severity and progression of the disease cannot be easily evaluated, and patients may not seek counseling for treatment.

Mucin-like protocadherin (MUPCDH), also known as mucin and cadherin-like protein, μ-protocadherin, and cadherin-related family member 5, is a novel member of the cadherin superfamily^10^. The *MUPCDH* gene, containing 15 exons, is located on chromosome 11p15.5; it encodes a mucin-like 110-kDa type I transmembrane protein^11^. Classical cadherins contain five extracellular cadherin repeats, and a conserved cytoplasmic region that interacts with the actin cytoskeleton via catenins. Functionally, the *MUPCDH* gene product is reported to be involved in calcium-dependent cell-cell adhesion, morphogenesis, cytoskeletal organization, and cell migration^12^,^13^. Although it has been reported that downregulation of *MUPCDH* expression is a common event in colorectal cancer^14^, its function in colorectal cancer is not established at present. Furthermore, the MUPCDH protein is expressed in various epithelial tissues and especially at the apical surface of differentiated proximal tubule epithelial cells of the kidney, where it may have additional roles unrelated to adhesion^11^. However, thus far, the precise functions of MUPCDH in the kidney are unknown.

Recently, environmental factors such as epigenetic alteration, as well as genetic modifiers, have been suggested to affect the pathogenesis of ADPKD^15^. It seems that environmental changes affect the epigenetic status in the body, and ultimately influence key signaling pathways that drive disease progression. In particular, DNA methylation is the most widely reported—and a stable—epigenetic marker^16^ that can heritably change gene expression without altering the DNA sequence^17^. In normal human cells, DNA methylation occurs at cytosine residues within CpG dinucleotides, which are mainly located in CpG islands (within the promoters of ~60% of genes), and is catalyzed by DNA methyltransferases (DNMTs). The establishment of aberrant DNA methylation patterns is associated with abnormal expression of certain genes, ultimately leading to diverse pathologies, such as cancer^18^. Interestingly, it appears that epigenetic modifications such as DNA methylation are early and tissue-specific events in tumorigenesis^19^, and therefore we could predict their application as potential prognostic markers in ADPKD, for renal cyst development, altered renal function, and ultimately disease progression. Several studies have in fact investigated DNA methylation markers in urine sediments from patients with bladder cancer, and reported that these epigenetic markers can be used for disease diagnosis^20^,^21^,^22^,^23^. In addition, epigenetic silencing is a potentially reversible alteration, which can be restored to the normal status by epigenetic therapy, unlike genetic mutation^24^.

In this study, we found that hypermethylation of *MUPCDH* promoter induces the transcriptional repression and increased cell proliferation in ADPKD. We suggested that urine methylation level of *MUPCDH* promoter region can be used as the novel epigenetic biomarker for prognosis of ADPKD.

## Results

### Aberrant hypermethylation of the *MUPCDH* gene promoter region in ADPKD

**Figure 1:**
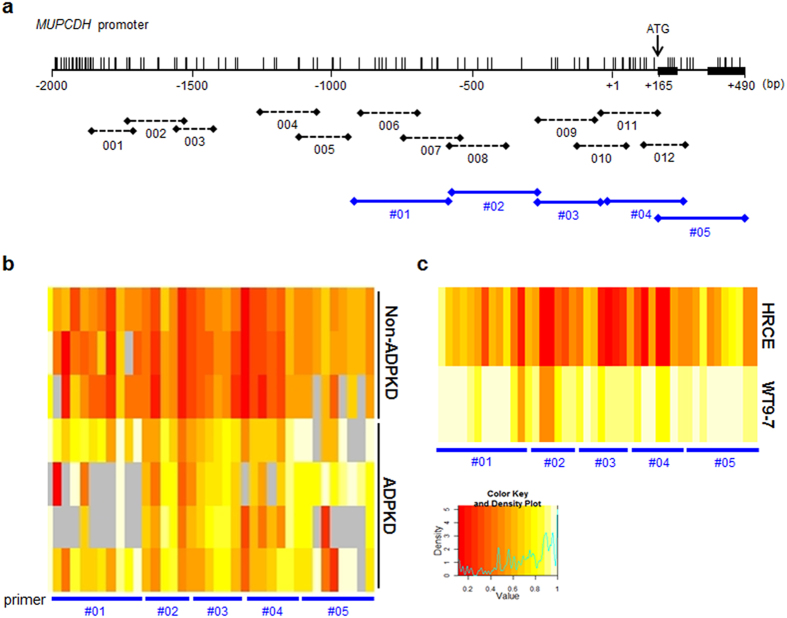
Hypermethylation of *MUPCDH* promoter region in ADPKD. (**a**) CpG sites on the *MUPCDH* promoter region are shown as vertical bars. The position of the translation start ATG is indicated by a black arrow. The exons are presented as thick black horizontal bars. Black horizontal dotted lines indicate the primer sites for methylation-sensitive high resolution melting analysis. Blue solid lines indicate the primer sites for the EpiTYPER® assay. (**b**) The DNA methylation status of the *MUPCDH* promoter CpG islands, covering approximately 1 kb upstream from the ATG, was quantitated by EpiTYPER® analysis in renal tissue and (**c**) renal epithelial cells. As shown in the EpiTYPER® heat map, hypermethylated CpG sites (yellow) were observed in ADPKD renal tissue (n = 4) and WT9-7 renal cystic epithelial cells, while CpG sites in non-ADPKD kidney tissue (n = 3) and human renal cortical epithelial cells were hypomethylated (red). *P* < 0.001.

In order to evaluate the DNA methylation pattern of genes related to cyst formation in ADPKD, methylated CpG island recovery assay-DNA sequencing (MIRA-seq.) analysis was carried out. The majority of genes, including *MUPCDH*, showed hypermethylation in their promoter region, which showed a negative correlation with the level of transcription (Supplementary Fig. S1a). The CpG island within the 5′ untranslated region (UTR) of the *MUPCDH* gene was significantly methylated in renal tissue from patients with ADPKD compared with non-ADPKD controls (Supplementary Fig. S1b). The DNA methylation pattern of the *MUPCDH* gene promoter region, which extends upstream from the translation start ATG for 2 kb to intron 1, was validated using methylation-sensitive high resolution melting (MS-HRM) analysis in individual clinical tissue samples and in renal cystic epithelial cells. Because of this analysis, the differentially methylated region (DMR) was identified as extending 1 kb upstream from the ATG of the *MUPCDH* gene (Supplementary Fig. S2). Following on from the MS-HRM analysis, the quantitative methylation status of the *MUPCDH* promoter was further confirmed using the EpiTYPER® assay. As expected, the upstream 1 kb region of the *MUPCDH* promoter was hypermethylated in both ADPKD renal tissue and WT9-7 ADPKD cyst-lining epithelial cells compared with non-ADPKD renal tissue and human renal cortical epithelial (HRCE) cells (Fig. 1). The data suggest that significant hypermethylation of the *MUPCDH* promoter CpG island is specifically observed in ADPKD.

### Downregulation of *MUPCDH* gene expression in ADPKD

**Figure 2:**
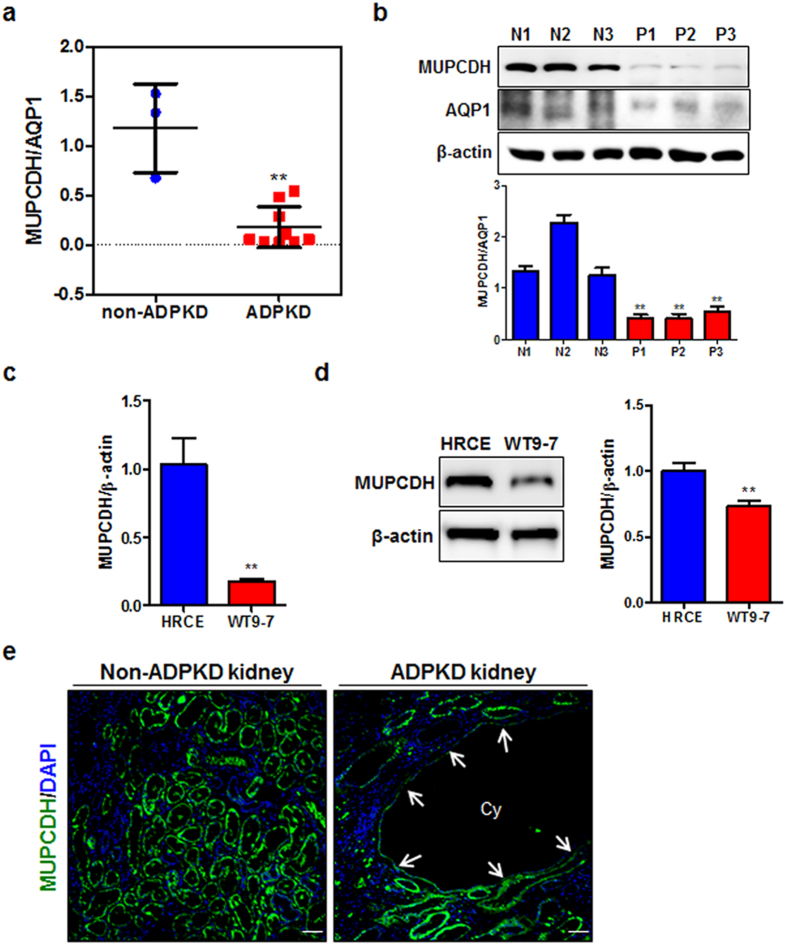
Downregulation of *MUPCDH* in ADPKD. (**a,b**) Gene expression levels were confirmed by real-time quantitative reverse transcription polymerase chain reaction (qRT-PCR) and western blot analyses, in non-ADPKD and ADPKD renal cortical tissue, and in (**c,d**) human renal cortical epithelial and renal cystic epithelial (WT9-7) cells. The band density in western blot analysis was measured using the MultiGauge software. The height of each bar represents the mean, and the error bars indicate ± SD. Aquaporin 1 (AQP1), which is a proximal tubule marker, and β-Actin were used as internal controls in real-time qRT-PCR and western blot analyses. Each experiment was performed in triplicate. ^*^P < 0.05; ^**^P < 0.01; ^***^P < 0.001. (**e**) Expression of MUPCDH protein (green) was analyzed by immunofluorescence in non-ADPKD and ADPKD renal tissues. The expression level of MUPCDH was dramatically reduced in the cyst-lining epithelial cells of ADPKD renal tissue, as indicated by arrows. Scale bars, 10 μm. Cy, cyst.

The expression levels of the *MUPCDH* mRNA and MUPCDH protein were confirmed using real-time quantitative reverse transcription polymerase chain reaction (qRT-PCR) and western blot analysis, respectively; significant repression of *MUPCDH* gene expression levels was observed in both ADPKD renal tissues and WT9-7 cells, compared with non-ADPKD renal tissues and HRCE cells (Fig. 2a–d). Interestingly, while MUPCDH protein is normally expressed apically along the brush border of the proximal convoluted tubule, its expression was dramatically suppressed in cyst-lining epithelial cells within ADPKD renal tissue (Fig. 2e). The data indicate that downregulation of the *MUPCDH* gene may be crucial to cyst development in ADPKD.

### Correlation between *MUPCDH* promoter hypermethylation and its transcriptional repression

**Figure 3:**
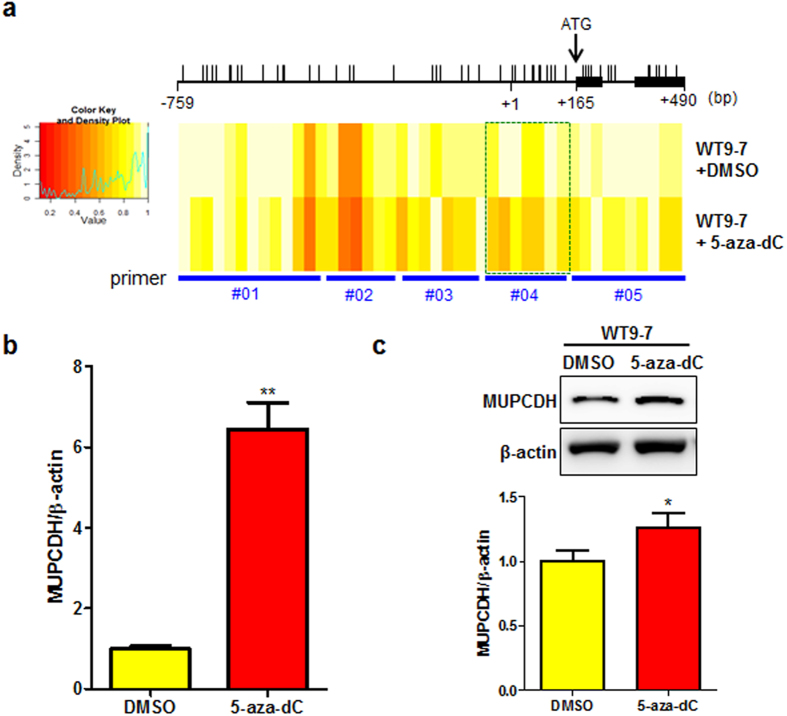
Restoration of the *MUPCDH* expression level by treatment with 5-aza-2′-deoxycytidine (5-aza-dC) in ADPKD cyst-lining epithelial cells. (**a**) The altered DNA methylation pattern in the *MUPCDH* promoter region was confirmed using the EpiTYPER® assay. As shown in the EpiTYPER® heat map, methylated CpG sites (yellow) were changed to unmethylated CpG sites (red) after treatment of WT9-7 cystic epithelial cells with 5-aza-dC (5 μM, 72 h). Green box indicate CpG sites within −543 and +228, which was more sensitive to 5-aza-dC, compared to those in other regions. The translation start ATG is indicated by a black arrow. *P* < 0.05. (**b**) Altered mRNA and protein expression levels upon treatment of WT9-7 cells with 5-aza-dC were confirmed by real-time quantitative reverse transcription polymerase chain reaction (qRT-PCR) analysis, and (**c**) western blot analysis. The band density was measured using the MultiGauge software. The height of each bar represents the mean, and the error bars indicate ± SD. β-Actin was used as an internal control in real-time qRT-PCR and western blot analyses. Each experiment was performed in triplicate. ^*^P < 0.05; ^**^P < 0.001.

In order to establish whether the restoration of the DNA methylation level affects the regulation of *MUPCDH* gene expression, WT9-7 cells were treated with the demethylating agent, 5-aza-2′-deoxycytidine (5-aza-dC). Following treatment of the cells with 5-aza-dC, the DNA methylation level of the *MUPCDH* promoter region was decreased (Fig. 3a). In particular, as shown in Fig. 3a, specific promoter region presented by green dotted box was more sensitive to 5-aza-dC, compared to those in other regions. The data indicate that this particular region may be more critical for the regulation of *MUPCDH* transcription in the presence of DNA methylation. Furthermore, the mRNA and protein levels expressed from the *MUPCDH* gene were restored by demethylation of the promoter region in ADPKD cystic epithelial cells (Fig. 3b,c). Although demethylation induced by 5-aza-dC in WT9-7 cells was mean 73% within the green dotted box region, which was not similar compared with those of HRCE cells (~26%), it was sufficient to restore suppressed *MUPCDH* expression level (Supplementary Fig. S3). This *in vitro* result implies that reduced methylation of the *MUPCDH* promoter CpG islands can restore its expression at both the mRNA and protein level in ADPKD.

### Association between *MUPCDH* promoter methylation level in urine samples and renal progression in human ADPKD

**Figure 4:**
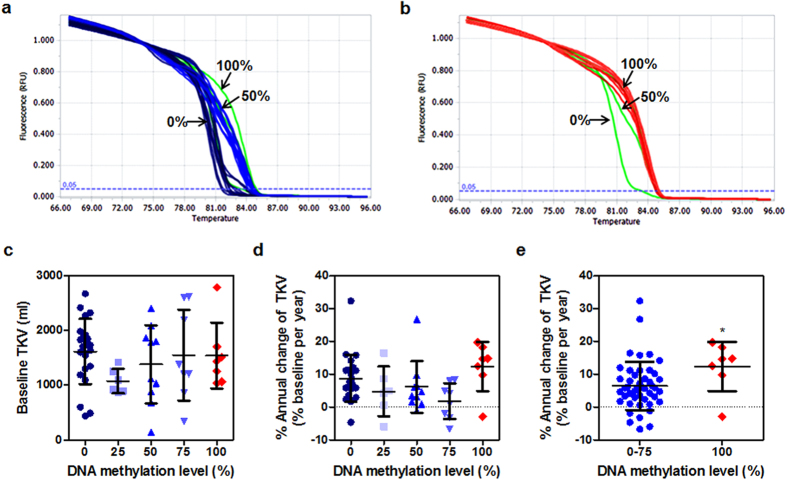
Correlation between hypermethylation of *MUPCDH* promoter and the increased rate of total kidney volume (TKV) change in ADPKD. (**a,b**) Methylation-sensitive high resolution melting (MS-HRM) analysis was carried out using urine sediment genomic DNA from patients with autosomal dominant polycystic kidney disease (ADPKD). Bisulfite converted human control DNA (0, 50, 100% methylated) was used as quantification standards for MS-HRM analysis (green lines). Based on control DNA, ADPKD patients were classified into five groups as a methylation level of *MUPCDH* promoter region. Dark blue and blue lines indicate unmethylated and hemi-methylated samples. Red line showed fully methylation pattern. (**c**) Baseline TKV and (**d**) rate of annual change of TKV according to *MUPCDH* promoter methylation level were shown. (**e**) When the subjects were grouped into 0–75% (n = 46) vs. 100% (n = 7) methylation level, percent change in TKV per year was significantly faster in 100% group than 0–75% (median 14.6% vs. 5.9% per year, ^*^P = 0.019 by Mann-Whitney test).

To evaluate the potential as a prognostic biomarker of ADPKD, DNA methylation levels in the proximal promoter region of the *MUPCDH* gene were measured in random urine samples (n = 53) from patients with ADPKD using MS-HRM analysis. We classified the subjects into five groups according to methylation level of the *MUPCDH* promoter (Fig. 4a,b), and compared the total kidney volume (TKV) and estimated glomerular filtration rate (eGFR) among groups. The mean age of subjects was 49 ± 9.3 years and they were 53% male. Baseline TKV and eGFR were 1491 ± 632.5 ml and 60.8 ± 18.1 ml/min per 1.73 m^2^. The mean interval between the baseline and final value was 21 months. There was no significant difference in baseline TKV (Fig. 4c), nor the rate of annual change of TKV (Fig. 4d) according to *MUPCDH* promoter methylation level. However, when the subjects were grouped into 0–75% and 100% methylation groups, the percent annual change in TKV was significantly faster in the 100% methylation group than the 0–75% group (median 14.6% vs. 5.9% per year, *P* = 0.019 by Mann-Whitney test) (Fig. 4e). In contrast, no significant trend was observed in baseline kidney function or annual change in eGFR (Supplementary Fig. S4). This result implies that the fully methylated *MUPCDH* promoter may reflect the progression of cyst growth in ADPKD.

### Transcription factor AP-2α involved in the promoter activity of the *MUPCDH* gene

**Figure 5:**
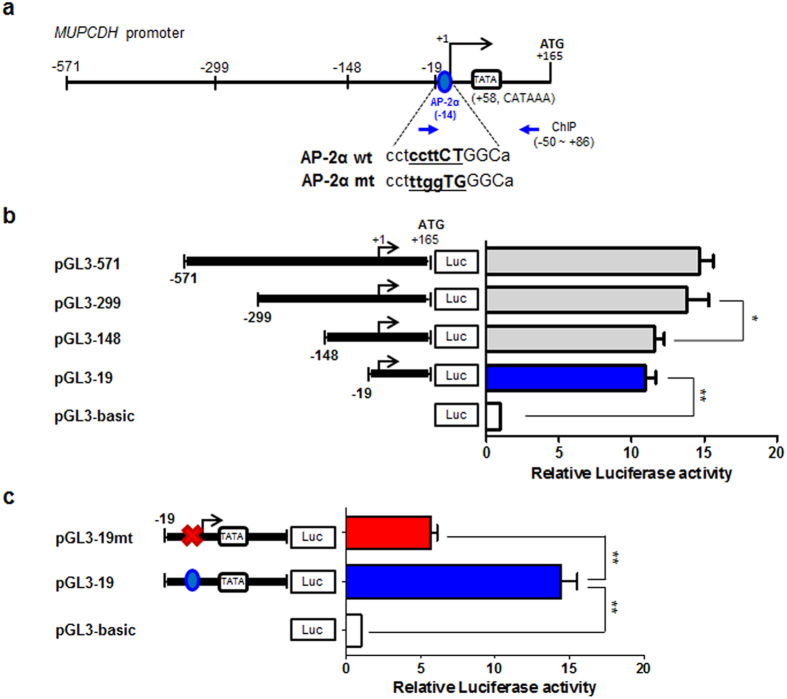
The proximal region of the *MUPCDH* promoter responsible for active transcription. (**a**) Scheme for generating serial deletion fragments of the *MUPCDH* promoter region (from −571 to +165). The transcription factor AP-2α binding site (−14) is indicated by a blue circle. Sequences that were mutated by site-directed mutagenesis are indicated in underlined bold type. Primers for the chromatin immunoprecipitation assay are indicated by blue arrows. (**b**) Luciferase assays of four *MUPCDH* promoter region constructs were carried out using HEK293T cells. The ratio of *Renilla* luciferase to Firefly luciferase was calculated for each experiment, and the values from triplicate experiments were averaged. The mean value for each test construct was normalized to the activity of the empty vector. Bars represent the mean of the normalized values with error bars indicating the range. (**c**) Site-directed mutagenesis of the transcription factor AP-2α binding sequence induced a significant repression of the luciferase activity of the pGL3-19 construct. ^*^P < 0.01; ^**^P < 0.001.

**Figure 6:**
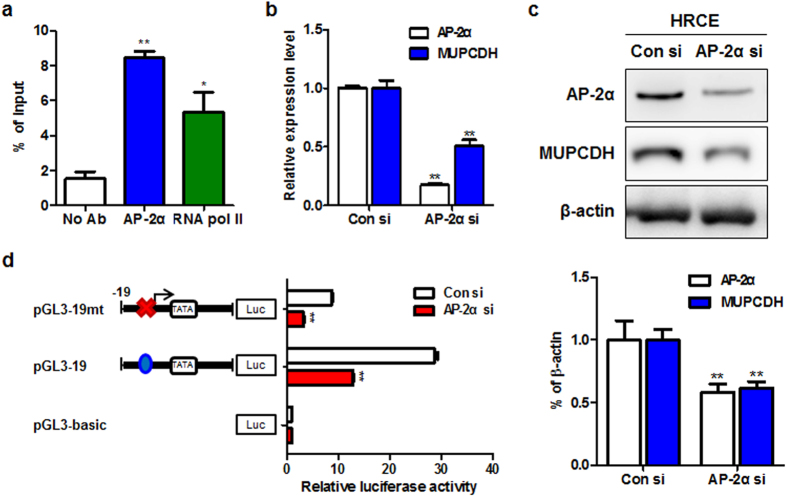
The regulation of *MUPCDH* transcription via transcription factor AP-2α. (**a**) As assayed by chromatin immunoprecipitation, together with quantitative reverse transcription polymerase chain reaction (qRT-PCR), AP-2α was highly enriched in the *MUPCDH* promoter region of human renal cortical epithelial cells. RNA polymerase II was used as a positive control. (**b**,**c**) Knockdown of AP-2α decreased the mRNA and protein expression levels of *MUPCDH*, as confirmed by real-time qRT-PCR and western blot analyses, respectively. The band density was measured using the MultiGauge software. (**d**) The luciferase activity was measured following knockdown of AP-2α in HEK293T cells. The ratio of *Renilla* luciferase to Firefly luciferase was calculated for each experiment, and the values from triplicate experiments were averaged. The mean value for each test construct was normalized to the activity of the empty vector. Bars represent the mean of the normalized values with error bars indicating the range. Each experiment was performed in triplicate. ^*^P < 0.01; ^**^P < 0.001.

To further investigate which specific region within the *MUPCDH* gene promoter is critical to the regulation of gene transcription, serial deletion constructs of the *MUPCDH* gene 5′ UTR were generated by cloning appropriate PCR-amplified fragments (Fig. 5a) into the pGL3-basic luciferase reporter vector, and transfected into human embryonic kidney HEK293T cells. As shown in Fig. 5b, the pGL3-299 and pGL3-571 constructs showed higher luciferase activity than the pGL3-148 construct, but this was only a 1.5-fold enhancement compared with the pGL3-19 construct. Importantly, the promoter activity of pGL3-19 was sufficient to activate expression of the reporter gene (Fig. 5b), indicating that this region of the promoter has a critical role in transcriptional regulation of the *MUPCDH* gene. Promoter analysis using programs such as those in the TRANSFAC® database indicated that the consensus sequence of transcription factor AP-2α is located at −14 to −2 in the *MUPCDH* gene promoter (Fig. 5a). To verify that the AP-2α binding site is necessary to promote *MUPCDH* transcription, the predicted consensus sequence for AP-2α binding within the pGL3-19 construct was mutated by site-directed mutagenesis, and the effect of this mutation on promoter activity was measured in the luciferase assay. Mutation of the AP-2α binding element significantly reduced the expression of the reporter (Fig. 5c). These data support the conclusion that pGL3-19 construct may be required for the regulation of *MUPCDH* promoter activity. Moreover, the results of a chromatin immunoprecipitation-qRT-PCR (ChIP-qPCR) assay for AP-2α binding showed significant enrichment of RNA polymerase II and AP-2α on the *MUPCDH* proximal promoter sequence in HRCE cells which have a high level of *MUPCDH* gene expression (Fig. 6a). Whereas, other predicted possible transcription factors such as Sp1, WT1, Stat5 and HLTF, did not show significant enrichment even though their binding sites are overlapped each other within the proximal promoter region of *MUPCDH* (Supplementary Fig. S5), supporting the specificity of the AP-2α into the *MUPCDH* promoter. Transfection of *AP-2α*-targeted short interfering RNA (siRNA) induced a reduction in the level of *MUPCDH* expression (Fig. 6b,c). To further confirm the interaction between AP-2α and the *MUPCDH* gene, the luciferase assay was carried out following transfection of the *AP-2α* siRNA into HEK293T cells. Upon knockdown of *AP-2α*, *MUPCDH* promoter activity was reduced to a much lower level in pGL3-19 construct (Fig. 6d). However, interestingly, overexpression of AP-2α in WT9-7 cells could not significantly increase the level of *MUPCDH* gene expression (Supplementary Fig. S6a). In contrast, this was not the case in normal renal epithelial cells, as the level of *MUPCDH* expression was increased upon overexpression of AP-2α in HRCE cells (Supplementary Fig. S6b). These data suggest that overexpressed AP-2α protein can bind to its binding element in the hypomethylated *MUPCDH* promoter in HRCE cells but not in WT9-7 cells, where the *MUPCDH* promoter is hypermethylated. Therefore, the data suggest that the activity of the *MUPCDH* promoter may be regulated by DNA methylation, as well as by the interaction of AP-2α with the promoter.

### Regulation of transcription factor AP-2α binding affinity by an altered DNA methylation pattern in the *MUPCDH* promoter

**Figure 7:**
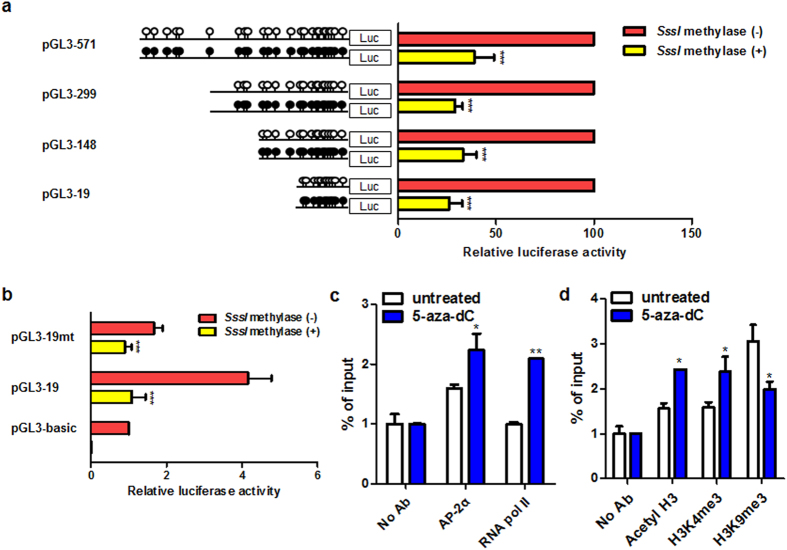
Proximal promoter activity repressed by an increased DNA methylation level. (**a**) Treatment of four luciferase constructs with methylase *SssI* induced a significant reduction of *MUPCDH* promoter activity *in vitro*. The luciferase activity following *SssI* methylase treatment is shown as a percentage of that of untreated constructs. (**b**) The luciferase activity of pGL3-19 wild-type and mutant constructs was assayed following treatment with *SssI* methylase. The ratio of *Renilla* luciferase to Firefly luciferase was calculated for each experiment and the values from triplicate experiments were averaged. The mean values of each test construct were normalized to the activity of the empty vector. (**c**,**d**) Chromatin immunoprecipitation-quantitative reverse transcription polymerase chain reaction was used to assay for RNA polymerase II, AP-2α, and histone modification marks in untreated and 5-aza-2′-deoxycytidine-treated (5 μM) WT9-7 cystic epithelial cells. Bars represent the mean of the normalized values with error bars indicating the range. Each experiment was performed in triplicate. ^*^P < 0.05; ^**^P < 0.01; ^***^P < 0.001.

An increased level of DNA methylation in the proximal promoter region of the *MUPCDH* gene may be a key factor in the regulation of *MUPCDH* gene transcription. To investigate methylation-associated inactivation of the *MUPCDH* gene in ADPKD, serial deletion constructs for use in the luciferase assay were treated with *SssI* methylase *in vitro*, and then transfected into HEK293T cells; increased methylation of promoter constructs induced significant repression of overall promoter activity in comparison with untreated constructs (Fig. 7a). In particular, hypermethylation of the proximal promoter region of the *MUPCDH* gene (corresponding to construct pGL3-19) inhibited promoter activity, similarly to the mutant pGL3-19 construct (Fig. 7b). Moreover, the enrichment of RNA polymerase II and AP-2α were increased by treatment of WT9-7 cells with 5-aza-dC (Fig. 7c). Intriguingly, the enrichment of active histone modification marks such as acetyl-histone H3 (acetyl-H3) and trimethyl histone H3 (Lys4) (H3K4 me3) were increased, while the inactive trimethyl histone H3 (Lys9) (H3K9 me3) mark was decreased, after treatment of WT9-7 cells with 5-aza-dC (Fig. 7d). These data indicate that the methylation pattern of the proximal promoter region of the *MUPCDH* gene may affect the binding affinity of AP-2α and the transcription level associated with histone modification marks.

### Inhibition of cell proliferation by restoration of the level of *MUPCDH* expression

**Figure 8:**
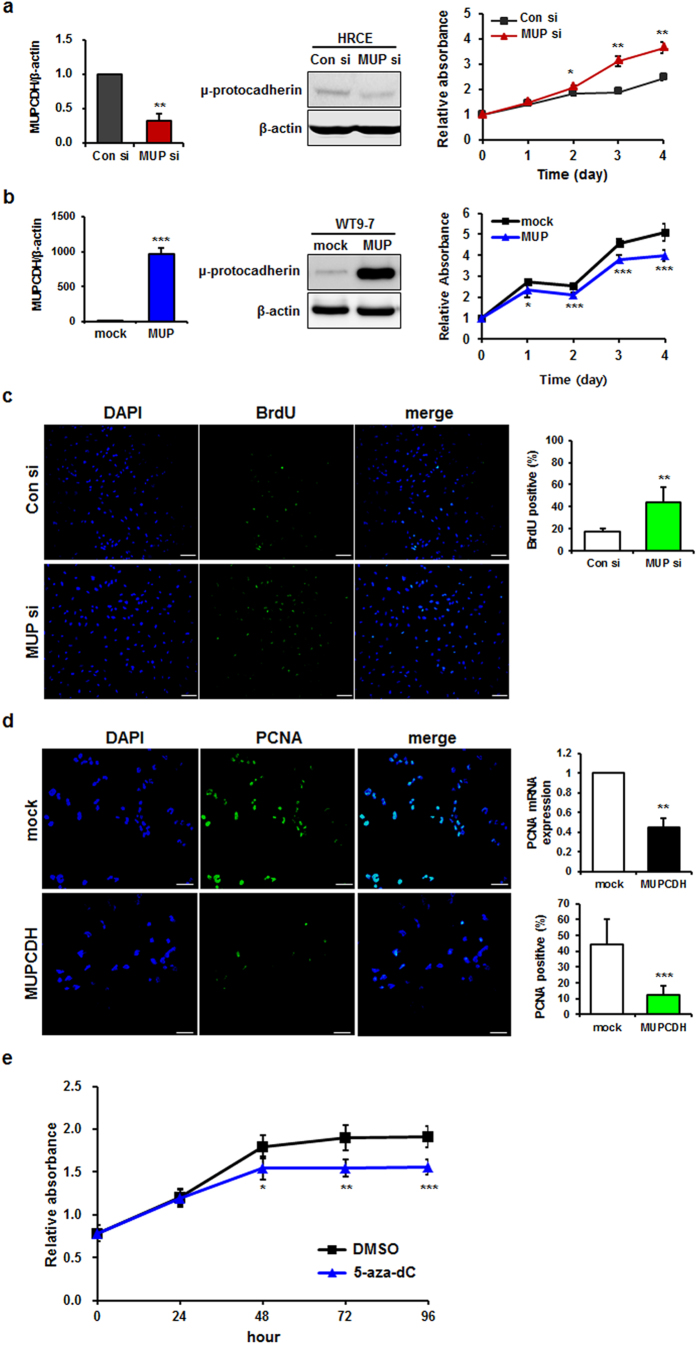
Inhibitory effect of MUPCDH on cell proliferation. (**a**) Knockdown of the *MUPCDH* gene increased cell survival in human renal cortical epithelial (HRCE) cells. (**b**) Overexpression of the *MUPCDH* gene decreased cell growth of WT9-7 cells. (**c**) 5-bromo-2′-deoxyuridine (BrdU) incorporation assays were carried out using HRCE cells that had been transfected with *MUPCDH* short interfering RNA (siRNA) or control siRNA. (**d**) Proliferating cell nuclear antigen (PCNA) staining carried out using HEK293T cells that overexpressed the *MUPCDH* gene. The level of PCNA expression was also measured by real-time quantitative reverse transcription polymerase chain reaction (qRT-PCR). β-Actin was used as an internal control in real-time qRT-PCR assays. (**e**) Cell proliferation rate was measured using a cell proliferation assay kit (WST-1) following treatment of WT9-7 cells with 5-aza-2′-deoxycytidine (5 μM). The experiment was performed in triplicate. ^*^P < 0.05; ^**^P < 0.01; ^***^P < 0.001.

To investigate how silencing of the *MUPCDH* gene affects the progression of cyst formation due to elevated cell proliferation in ADPKD, knockdown or overexpression of the *MUPCDH* gene was induced in HRCE and WT9-7 cells. When the *MUPCDH* gene was downregulated in HRCE cells, cell proliferation was increased (Fig. 8a). In contrast, overexpression of *MUPCDH* in WT9-7 cells induced the suppression of cell growth (Fig. 8b). To further confirm the anti-proliferative effect of MUPCDH, the 5-bromo-2′-deoxyuridine (BrdU) incorporation assay was carried out following transfection of *MUPCDH*-targeted siRNA into HRCE cells. As expected, the incidence of BrdU-positive cells was greater in cells transfected with the *MUPCDH* siRNA than in control siRNA-transfected cells (Fig. 8c). In addition, overexpression of the *MUPCDH* gene significantly reduced both the level of proliferating cell nuclear antigen (PCNA) mRNA and the number of PCNA-positive cells (Fig. 8d). Intriguingly, treatment with 5-aza-dC induced the inhibition of cell proliferation, in agreement with the data for *MUPCDH* overexpression (Fig. 8e). Taken together, these results indicate that the *MUPCDH* gene may be involved in abnormal proliferation of renal epithelial cells, and that restoration of DNA methylation can regulate the anti-proliferative property of MUPCDH.

## Discussion

Hypermethylation is a common event in the silencing of tumor suppressor genes in most cancer types^25^. Although ADPKD pathogenesis is also associated with epigenetic alterations such as histone modification and dysregulation of micro RNAs^26^,^27^, epigenetic biomarkers for the diagnosis or prognosis of ADPKD have not yet been established. In our previous study, we suggested the potential role of epigenomic alteration, especially DNA methylation, in cystogenesis of ADPKD, which is a genetic disorder^15^. In this study, we determined that a novel protocadherin gene associated with cyst growth in ADPKD is regulated by DNA methylation of its promoter region. We also examined the relationship between hypermethylation of the *MUPCDH* promoter and the clinical phenotypes of ADPKD patients. Intriguingly, in urine samples of patients with ADPKD, the fully methylated *MUPCDH* promoter was associated with faster kidney volume progression, suggesting that it may be a valuable prognostic biomarker of cyst growth in ADPKD. In particular, MS-HRM analysis is a simple and useful technique for simultaneously detecting DNA methylation patterns at multiple CpG sites using a small quantity of genomic DNA and for various samples. However, our analysis had the following limitations: 1) the sample size was relatively small, especially that of the 100% methylation group; 2) the average interval between the baseline and final value was 21 months years; and 3) MUPCDH is mainly expressed in proximal tubular cells, whereas cysts in ADPKD originate from both proximal and distal tubules, explaining a weak correlation with disease progression. Independent sets of serial samples with longitudinal data, including total kidney volume and renal function, are required to establish the validity of *MUPCDH* as a reliable ADPKD biomarker.

As reported in several previous studies, MUPCDH is a novel member of the cadherin superfamily that includes E-cadherin, and may play a role in calcium-dependent cell adhesion^28^. However, as MUPCDH is enriched at the apical surface of renal proximal tubule epithelial cells^11^, it may have additional functions that affect renal physiology and pathology. Our results suggest a novel function of MUPCDH, i.e., its anti-proliferative effect in the kidney. The loss of this function is important for the process of cyst formation, consistent with the significantly repressed level of MUPCDH expression across the cyst-lining epithelial cells in ADPKD renal tissue samples.

In a previous study, the *MUPCDH* gene was shown to be a target of the Cdx2 transcription factor in the colon cancer^29^. However, it is possible that another transcription factor is also required for the regulation of *MUPCDH* transcription, because the expression of *MUPCDH* and Cdx2 did not completely overlap in colon cancer cells, and the transcriptional regulation of *MUPCDH* expression by Cdx2 depended on the cell line^29^. We identified another transcription factor, AP-2α, that is specifically associated with the regulation of *MUPCDH* transcription. AP-2α has well-known roles in cell growth, normal morphogenesis, and apoptosis in mammalian cells^30^,^31^. A recent study also revealed that the AP-2α protein is overexpressed in nasal polyp epithelia compared to normal nasal mucosa, and promotes expression of the *MUC8* gene, which is an important mucin gene, and the major component of mucus^32^. Moreover, AP-2α binds to CpG islands in the promoter regions of various genes and is involved in gene transcription^33^.

More intriguingly, our data showed that transcription of the *MUPCDH* gene, and the functions of its gene product at the cellular level are regulated by promoter methylation. Treatment of renal cyst-lining epithelial cells with the DNA demethylating agent, 5-aza-dC, induced the re-expression of the *MUPCDH* gene; these results are in agreement with previous studies showing that DNA methylation is responsible for gene silencing^34^. Additionally, our data suggested an explanation for the importance of differential patterns of DNA methylation in promoter regions for transcriptional regulation. DNA methylation may influence gene transcription via two major mechanisms. First, the association between the methylated DNA and methyl-CpG-binding domain (MBD) proteins recruits additional proteins to the locus, such as histone deacetylases and other chromatin remodeling proteins, and ultimately leads to the alteration of the chromatin structure. Second, methylated cytosine residues interfere with the binding between transcription factors and their binding sites in the promoter region^35^. Because many transcription factor-binding sites do not include CpG dinucleotides, the second mechanism has been thought to have minimal transcriptional effects. However, we demonstrated that AP-2α is another transcriptional regulator of the *MUPCDH* gene. Although the relatively low efficiency of AP-2α transfection into WT9-7 cells compare with HRCE cells may be responsible for the lack of response in *MUPCDH* transcription level, it is also possible that the methylation of CpG sites in close proximity to the binding site changes the DNA double helix configuration or interferes with cofactor binding, and indirectly inhibits AP-2α binding. To our knowledge, this is the first report to identify a critical regulatory region within the *MUPCDH* promoter, and its high correlation with the DNA methylation pattern in the kidney. Further studies are needed to clarify the possible mechanism of the reduced binding affinity between the transcription factor and promoter sequence, including identification of cofactors that bind AP-2α.

Transcriptionally active promoters have a chromatin structure that is enriched for active histone marks such as acetyl-H3 and H3K4 me3, and enrichment of these active histone marks was induced following treatment of cyst-lining epithelial cells with the DNA demethylating agent, 5-aza-dC. In contrast, a reduction in H3K9 me3, which is an important modification for transcriptional repression, was observed in 5-aza-dC-treated cyst-lining epithelial cells. These data indicate that histone modification is involved in the aberrant DNA methylation pattern of the *MUPCDH* promoter region. However, it is still unclear how histone modification and DNA methylation cooperate to achieve gene silencing. It has been reported that the link between DNA methylation and histone modification might be partially mediated by MBD proteins, which recruit histone deacetylases to methylated DNA^36^. In addition, 5′-methylcytosine can also recruit dimethylated H3K9 (H3K9 me2), which is a repressive mark, by interacting with both histone methyltransferase G9a and DNMT1, and can interfere with H3K4 me3 interactions^37^,^38^. These interactions help explain how DNA methylation affects chromatin structure and gene silencing. Therefore, they should be validated in order to elucidate the relationship between DNA methylation and histone modification in the transcriptional regulation of *MUPCDH*.

In conclusion, our study shows that hypermethylation of a CpG island in the promoter region of the *MUPCDH* gene regulates its transcriptional level and affects the proliferative capacity of renal epithelial cells. These data imply that the differential methylation pattern of the *MUPCDH* promoter in non-ADPKD and ADPKD renal tissues is a potential target for a novel epigenetic therapeutic reagent, such as a demethylating agent, in ADPKD. Encouraged by these results, we plan to confirm that the methylation status obtained from urinary genomic DNA can actually affect cyst development and cause loss of renal function in ADPKD.

## Methods

### Renal tissue and urine samples from subjects with ADPKD

Renal cyst tissue was obtained from patients with ADPKD undergoing nephrectomy. As controls, non-ADPKD renal tissue specimens were obtained from patients undergoing surgery for clear cell renal cell carcinoma; malignant cell infiltration was excluded by histology. These tissues were all used in our previous study^15^. First or second, freshly voided urine samples were collected and stored at −70 °C from 53 subjects with ADPKD. Clinical information such as gender, age, eGFR, and kidney volume was obtained. Estimated GFR was calculated by using MDRD formula based on serum creatinine traceable to isotope dilution mass spectrometry. All the subjects were ascertained to have mild to moderate renal insufficiency (chronic kidney disease stage 2 ~ 3 b). Computed tomography was performed every two year and the TKV was measured by modified ellipsoid method. Rate of change in eGFR and TKV was calculated by subtracting final value from baseline, then dividing by baseline value, resulting percent annual change (% per year). The study was approved by the Institutional Review Board of Seoul National University Hospital (H-0701–033–95 and H-0901–046–269). Written informed consent was obtained from all subjects in accordance with the Declaration of Helsinki.

### Treatment of cell lines with 5-aza-dC and siRNA

HEK293T and WT9-7 cells^39^ were cultured in Dulbecco’s modified Eagle’s medium (Welgene, Dalseo-gu, Korea) supplemented with 10% (v/v) fetal bovine serum (FBS), and penicillin-streptomycin. For use in control experiments, HRCE cells were obtained from Clonetics (San Diego, CA, USA). HRCE cells were cultured in renal epithelial cell growth medium (Clonetics) with the following supplements: 0.5% FBS, human transferrin (10 mg/ml), hydrocortisone (0.5 mg/ml), insulin (5 mg/ml), triiodothyronine (5 × 10^−12^ M), epinephrine (0.5 mg/ml), epidermal growth factor (10 mg/ml), and penicillin-streptomycin. Cells were grown in a humidified atmosphere at 37 °C in 5% CO_2_ and 95% air. The cells were treated with 5 μM 5-aza-dC (Sigma-Aldrich, St Louis, MO, USA) for 72 h. The medium was changed daily thereafter, and fresh 5-aza-dC added as before. For transient transfection with siRNA, siRNA duplex oligoribonucleotides targeting either *MUPCDH* or *AP-2α* were obtained from Santa Cruz Biotechnology (Dallas, TX, USA). The siRNA was transfected into either HRCE or HEK293T cells using Lipofectamine^TM^ RNAiMAX (Invitrogen, Carlsbad, CA, USA), according to the manufacturer’s instructions. Subsequent experiments were performed 24–48 h after transfection.

### Bisulfite treatment and DNA methylation analysis

Genomic DNA was isolated from human renal tissue and renal cells, before and after treatment with DNMT inhibitors, using the NucleoSpin® TriPrep Extract kit (MACHEREY-NAGEL, Düren, Germany), according to the manufacturer’s protocol. Genomic DNA was extracted from urine specimens of 1.75 ml using the Urine DNA Isolation Kit (Norgen Biotek Corp., Thorold, ON, Canada), according to the manufacturer’s protocol. The EZ DNA Methylation-Gold^TM^ kit (Zymo Research, Orange, CA, USA) was used for bisulfite treatment of genomic DNA, according to the manufacturer’s instructions. DNA methylation patterns were validated in two steps, using MS-HRM and EpiTYPER® analyses. For MS-HRM analysis, primers covered the region extending for 2 kb from the translation start ATG upstream to intron 1 (Fig. 1 and Supplementary Table S1). PCR amplification was carried out in a total volume of 20 μl, containing the following: 10× Buffer, 1.65 mM MgCl_2_, 200 μM each of the four dNTPs, 500 nM each primer, 0.04 U HotStarTaq DNA polymerase (QIAGEN, Hilden, Germany), ResoLight dye (Roche, Branchburg, NJ, USA), and 10 ng of bisulfite-modified template. High-resolution melting was analyzed using the LightCycler® 96 Instrument (Roche). EpiTYPER® primers and the *MUPCDH* promoter region extending 1 kb upstream from the ATG are shown in Fig. 1 and Supplementary Table S2. For *in vitro* transcription, reverse primers were tagged with the bacteriophage T7 RNA polymerase promoter sequence, and 10-mer sequences were added to the forward primer for balance. Bisulfite-treated genomic DNA was amplified by PCR as follows: 94 °C for 15 min; 45 cycles of 94 °C for 20 sec, 62 °C for 30 sec, and 72 °C for 1 min; followed by 72 °C for 3 min. Unincorporated dNTPs left over following amplification were neutralized by incubation at 37 °C for 20 min with shrimp alkaline phosphatase (SAP) and inactivated at 85 °C for 5 min. *In vitro* RNA transcription, with an RNase A cleavage reaction cocktail containing 0.64× T7 polymerase buffer, 3.14 mM dithiothreitol, T7 R&DNA™ polymerase, 0.09 mg/ml RNase A, T- or C-cleavage mix, and SAP-treated PCR product, generated transcripts that had undergone enzymatic base-specific cleavage. The molecular mass of fragments was determined by matrix-assisted laser desorption/ionization mass spectrometry, and use of the EpiTYPER^TM^ software (Sequenom, San Diego, CA, USA), which automatically generates a report that contains quantitative information for each fragment analyzed.

### Total RNA extraction and real-time qRT-PCR

Total RNA was extracted using the NucleoSpin^®^ TriPrep Extract kit (MACHEREY-NAGEL), according to the manufacturer’s protocol. RNA (2 μg, isolated as described above) was reverse-transcribed using M-MLV reverse transcriptase (Promega, Madison, WI, USA), 100 nM oligo-dT, 1 mM each of the four dNTPs, and RNase inhibitor. The following primers were used for real-time qPCR: human *MUPCDH* (forward 5′-GCCGACCTTGCCCGCTACTCA-3′, and reverse 5′-CGCCACGGTGCCACGATACAG-3′), human *Aquaporin 1* (*AQP1*) (forward 5′-TATGCGTGCTGGCTACTACCGA-3′, and reverse 5′-GGTTAATCCCACAGCCAGTGTAG-3′), and human *ACTB* that encodes β-actin (forward 5′-AAGGCCAACCGCGAGAAGAT-3′, and reverse 5′-CCAGAGGCGTACAGGGATAGCAC-3′). Real-time qPCR was carried out using the quantitative SYBR® kit (JMC R&D, Mansfield, QLD, Australia) in the Rotor-Gene 3000® (Corbett Robotics, San Francisco, CA, USA), as described in the manufacturer’s instructions. PCR cycling conditions were as follows: 15 min at 95 °C, then 40 cycles of 10 sec at 95 °C, 15 sec at 60 °C, and 20 sec at 72 °C.

### Western blot analysis

Equal amounts of protein were analyzed in duplicate by SDS-PAGE. The following monoclonal antibodies were used: anti-MUPCDH (anti-μ-protocadherin, C-20; Santa Cruz Biotechnology), anti-AP-2α (Santa Cruz Biotechnology), anti-AQP1 (Santa Cruz Biotechnology), and anti-β-actin (Bethyl Laboratories, Montgomery, TX, USA). Immunoreactive proteins were detected by horseradish peroxidase-conjugated secondary antibodies and enhanced chemiluminescence reagents (GE Healthcare Bio-Sciences, Pittsburgh, PA, USA). All immunoblots were performed with triplicate and visualized by LAS image analyzer (Fujifilm, Tokyo, Japan). The band density was quantified using MultiGauge (Fujifilm).

### Immunohistofluorescence

Paraffin-embedded sections of renal tissue derived from patients with ADPKD and controls were melted in a 60 °C oven and deparaffinized in three changes of xylene, followed by rehydration in a graded ethanol series. Sections were heated at 100 °C for 15 min in 0.01 M citric acid (pH 6.0) for antigen retrieval, and blocking was performed for 1 h at room temperature with blocking solution (VECTASTAIN® ABC kit, Vector Laboratories Inc. Burlingame, CA, USA), followed by overnight incubation at 4 °C with anti-MUPCDH primary antibody (anti-μ-protocadherin, C-20; Santa Cruz Biotechnology). Incubation with 4′,-6-diamidino-2-phenylindole was carried out in order to stain cell nuclei. Fluorescent signals were visualized using a fluorescence microscope (Olympus IX-81).

### Plasmid construction

Serial deletion fragments of the *MUPCDH* promoter region (−571, −299, −148, −19/+1; +1 being the transcription start site) were amplified by PCR and cloned into a pGL3-basic luciferase vector (Promega). A bacterial artificial chromosome clone (RP11; Invitrogen) was used as a template for PCR. A *NheI* site was added to the 5′-primer, and a *XhoI* site was added to the 3′-primer (these sites are underlined in the primer sequences). The sequences of the PCR primers used to generate serial deletion constructs were as follows: −571 forward 5′-ctagctagcGGGCCAATGAAAAGAGCA-3′, −299 forward 5′-ctagctagcGCCCCTGCCCTGCCCCTACC-3′, −148 forward 5′-ctagctagcGGGGTGGCCTTAGGAACTTTG-3′, −19 forward 5′-ctagctagcCTGCCCCTCCTTCTGGCAGTG-3′/+ 1 reverse 5′-ccgctcgagCTTGGCGGCTGTCACCTGGC-3′. The constructs were verified by sequencing of both DNA strands.

### Site-directed mutagenesis

The prediction of transcription factor binding sites was carried out using the TRANSFAC®database, and the promoter region of AP-2α was subjected to site-directed mutagenesis to identify the potential activator of the *MUPCDH* gene promoter. Site-directed mutagenesis was carried out using the QuikChange Site-Directed Mutagenesis Kit (Stratagene, La Jolla, CA, USA), according to the manufacturer’s instructions. The mutated sites are underlined as follows in the sequence corresponding to the forward primer for the −19 serial deletion fragment: 5′-CTGCCCCTCCTTCTGGCAGTG-3′→ 5′-CTGCCCCTTGGTGTGGCAGTG-3′.

### Transfection and luciferase reporter assays

For *in vitro* treatment with methylase *SssI*, cloned pGL3-constructs were redigested with the *NheI* and *XhoI* restriction enzymes, and then purified using the Zymoclean^TM^ Gel DNA Recovery Kit (Zymo Research). Purified insert fragments were subjected to *in vitro* methylation by methylase *SssI* in the presence of the methyl group donor, S-adenosylmethionine, and monitored by digestion with the *SmaI* enzyme. Methylated insert fragments were subsequently purified using DNA Clean & Concentrator^TM^-5 Kit (Zymo Research) and ligated with digested pGL3-basic vector. The ligation mixture was directly transfected into cells. For transfections, HEK293T cells were plated into 6-well plates at a density of 2 × 10^5^ per well, and luciferase constructs (2 μg of reporter gene plasmid and 50 ng of phRL-CMV normalizing plasmid) were transfected into the cells 1 day after seeding using FuGENE® HD transfection reagent (Roche), according to the manufacturer’s instructions. After transfection for 48 h, luciferase activity was measured by the Dual Luciferase Assay system (Promega) and relative luciferase activity was calculated by normalization to the *Renilla* luciferase activity of the phRL-CMV plasmid. All transfection experiments were carried out in duplicate and repeated at least three times.

### ChIP-qPCR

ChIP was performed as previously described^40^, using antibodies specific for RNA polymerase II (ab817; Abcam, Cambridge, UK), AP-2α (sc-184; Santa Cruz Biotechnology), H3K4 me3 (17-614; EMD Millipore, Billerica, MA, USA), H3K9 me3 (ab8898; Abcam), and H3Ac (06–599; EMD Millipore). Sequences of the *MUPCDH* promoter primers were as follows: forward 5′-CCCTGCACTCAGTCCAACTT-3′, and reverse 5′-TTCC TACCTCAGCGACCTTC-3′.

### Cell proliferation assays

To assess cell proliferation, WT9-7 and HRCE cells were seeded into 10-cm culture dishes at a density of 2.5 × 10^4^ per dish, and transfected with pCMV-SPORT6 (Open Biosystems, Huntsville, AL, USA) expressing MUPCDH, or *MUPCDH*-targeted siRNA, using FuGENE® HD transfection reagent (Roche) and Lipofectamine^TM^ RNAiMAX (Invitrogen), respectively. After 24 h, cells were plated at a density of 1,000 per well in 96-well plates, and grown for 1, 2, 3, and 4 days in standard culture medium. The proliferation of each cell line was measured by a colorimetric assay using the WST-1 Cell Proliferation Assay Kit (Roche), according to the manufacturer’s instructions. Absorbance of the converted dye was measured at a wavelength of 470 nm. In the BrdU incorporation assay, transfected cells were incubated in culture medium containing 10 μMBrdU (Sigma-Aldrich) for 2 h, and fixed with 4% v/v formaldehyde in phosphate-buffered saline (PBS). After washing with PBS, cells were treated with 2 M HCl for 20 min at 37 °C, and neutralized with 0.1 M sodium borate buffer for 15 min at 37 °C. Cells were permeabilized with 0.1% Triton X-100 for 15 min, and then washed with PBS. Finally, the cells were incubated with a mouse monoclonal anti-BrdU antibody (Sigma-Aldrich) at 4 °C overnight. For PCNA staining, transfected cells were fixed with 4% paraformaldehyde for 10 min at room temperature, and then washed three times with PBS. Cells were permeabilized with 0.2% Triton X-100 containing 5% v/v bovine serum albumin, and incubated with an anti-PCNA antibody (Santa Cruz Biotechnology) at 4 °C overnight. Immunofluorescence images for BrdU incorporation and PCNA staining were visualized using a confocal microscope (Olympus IX-70).

### Statistical analysis

Each experiment was repeated at least three times throughout the study. Data were reported as the mean ± SD. Statistical significance was examined using Student’s *t* test when only two groups were compared, otherwise one-way analysis of variance with Kruskal-Wallis test or Mann-Whitney test as appropriate was carried out, using the GraphPad Prism 5.0 program (GraphPad Software, San Diego, CA, USA). *P* < 0.05 was considered as significant.

## Additional Information

**How to cite this article**: Mi Woo, Y. *et al.* Epigenetic silencing of the MUPCDH gene as a possible prognostic biomarker for cyst growth in ADPKD. *Sci. Rep.* **5**, 15238; doi: 10.1038/srep15238 (2015).

## Supplementary Material

Supplementary Information
