# Supplementary material for: Epigenetic silencing of the MUPCDH gene as a possible prognostic biomarker for cyst growth in ADPKD
Source: Sci Rep. 2015 Oct 14;5:15238. doi: 10.1038/srep15238 (PMC4604459; doi:10.1038/srep15238)

Scientific Reports

Supplementary Information

Epigenetic silencing of the MUPCDH gene as a possible prognostic biomarker for cyst growth in ADPKD

Yu Mi Woo[1], Yubin Shin[1], Jung-Ah Hwang[2], Young-Hwan Hwang[3], Sunyoung Lee[1], Eun Young Park[1], Hyun Kyung Kong[1], Hayne Cho Park[4], Yeon-Su Lee[2,*] and Jong Hoon Park[1,*]

[1]Department of biological science, Sookmyung Women's University, Seoul, 140-742, Korea
[2]Branch of Cancer Genomics, Research Institute, National Cancer Center, Goyang Gyeonggi-do, Korea
[3]Department of Internal Medicine, Eulji General Hospital, Seoul, 139-892, Korea
[4]Division of Nephrology, Armed Forces Capital Hospital, Seongnam, Korea
[*]Co-corresponding authors.

SUPPLEMENTARY TABLE AND FIGURE LEGENDS

Supplementary Table S1 Primer sequences for methylation-sensitive high resolution melting analysis

Supplementary Table S2 PCR Primer sequences for EpiTYPER

Supplementary Fig. S1 Methylated CpG island recovery assay-DNA sequencing analysis showing hypermethylation of the *MUPCDH* promoter region in autosomal dominant polycystic kidney disease (ADPKD). (A) The 50 selected genes, which are in the top 5% of those screened in terms of their DNA methylation level (P = 0.0259), showed a negative correlation between the methylation of their promoter region and their transcription level. The promoter region is defined as extending from 1 kb upstream to 500 bp downstream of the transcription start site. (B) The DNA methylation pattern of the *MUPCDH* gene and promoter region is presented in the UCSC genome browser (2009, GRCh37/hg19). The coding exons are shown as vertical bars and the 5′ and 3′ untranslated regions are shown as short vertical bars. The promoter region of the *MUPCDH* gene is indicated by the yellow box. The CpG island is located within the *MUPCDH* promoter region. Renal tissue from three patients with ADPKD showed a hypermethylation pattern in the *MUPCDH* coding region and promoter, when compared with three non-ADPKD renal tissue specimens.

Supplementary Fig. S2 Methylation pattern of the MUPCDH promoter region screened using methylation-sensitive high resolution (MS-HRM) analysis. The methylation level was evaluated by comparing the melting curves of each group. (A) Non-autosomal dominant polycystic kidney disease (ADPKD; blue line) and ADPKD (red line) clinical samples. (B) Human renal cortical epithelial (blue line) and renal cystic epithelial (WT9-7; red line) cells. The primer site that was used is indicated (by the number to the right upper end of each MS-HRM data). The MUPCDH proximal promoter region (extending 1kb upstream from the ATG, between the sites for MS-HRM primers no. 006 and 012) shows differential methylation in the non-ADPKD and ADPKD groups. The experiment was performed in triplicate.

SUPPLEMENTARY TABLE AND FIGURE LEGENDS

Supplementary Fig. S3 Fully methylated *MUPCDH* promoter (~100%) repress transcription, but ~70% methylation level can activate its transcription in ADPKD cystic epithelial cells. Each bar graph indicates the methylation level of *MUPCDH* promoter region within green dotted box in Fig. 3a as a result of EpiTYPER® assay.

Supplementary Fig. S4 Hypermethylation of *MUPCDH* promoter did not reflect the rate of renal failure in ADPKD. (A) Baseline kidney function and (B) the rate of change in kidney function did not show significant difference among the MUPCDH methylation groups. A dot depicts each subject and bar graphs represent median and interquartile range. eGFR, estimated glomerular filtration rate.

Supplementary Fig. S5 Only AP-2α binds to the *MUPCDH* promoter (pGL3-19 construct). (A-B) Chromatin immunoprecipitation-quantitative reverse transcription polymerase chain reaction was performed for five possible transcription factors including Sp1, WT1, Stat5, HLTF, and AP-2α in HEK293T cells. RNA polymerase II was used as a positive control. Each experiment was performed in triplicate. *, $P < 0.01$; **, $P < 0.001$.

Supplementary Fig. S6 AP-2α overexpression is not sufficient to restore *MUPCDH* transcription in autosomal dominant polycystic kidney disease (ADPKD) cyst-lining epithelial cells. (A) AP-2α was overexpressed in WT9-7 cyst-lining epithelial cells and (B) human renal cortical epithelial cells, respectively. Both *AP-2α* and *MUPCDH* expression levels were confirmed using real-time quantitative reverse transcription polymerase chain reaction. The mRNA levels were normalized to *β-actin* mRNA, which acted as an internal loading control. *, $P < 0.01$; **, $P < 0.001$.

Supplementary Table S1. Primer sequences for MS-HRM

Primer ID	Start	PCR product size (bp)	Tm (℃)	GC%	Sequence
MUPCDH-HRM-001-F	154	165	60.79	50	GTTATAGGGTTTGGTGGGGAAG
MUPCDH-HRM-001-R	318		58.54	33	AAACCTAATCCAAACCATACTATTCTC
MUPCDH-HRM-002-F	294	180	56.16	32	GAATAGTATGGTTTGGATTAGGTTT
MUPCDH-HRM-002-R	473		57.91	41	TAAACCTCCAATAACCACCAAC
MUPCDH-HRM-003-F	454	128	59.89	33	TGGTGGTTATTGGAGGTTTAATTT
MUPCDH-HRM-003-R	581		59.66	27	ACCCAAAAAACAACCAAAAAAA
MUPCDH-HRM-004-F	761	174	58.53	32	GTTGGGAGTTGTTTGTGTTTATTTA
MUPCDH-HRM-004-R	934		56.64	24	ACAAAAAATAACCCTAATACCAAAA
MUPCDH-HRM-005-F	895	163	58.5	21	GTTTTTTATTTTTATTTTTGGTATTAGGG
MUPCDH-HRM-005-R	1057		60.26	43	AAACACTAACCACATAAACCCCC
MUPCDH-HRM-006-F	1126	176	59.98	45	GGAGGTGTTATTTGGGGTGTTA
MUPCDH-HRM-006-R	1301		56.5	31	AAAACTAAACTCAATATCCTAACCCT
MUPCDH-HRM-007-F	1273	155	58.34	32	TGTAGGGTTAGGATATTGAGTTTAGTTT
MUPCDH-HRM-007-R	1427		60.55	35	CAAAAACCCCCAACTCTAAAAAA
MUPCDH-HRM-008-F	1429	172	58.87	43	GGGGTTAATGAAAAGAGTAGGGT
MUPCDH-HRM-008-R	1600		59.99	43	CCTACCTACCCCAAAAACAAATC
MUPCDH-HRM-009-F	1766	179	56.3	42	GGTTAGGGAGGTAGGTTTTAGATA
MUPCDH-HRM-009-R	1944		59.83	33	CAAAAAACTCAAATCACAAACACC
MUPCDH-HRM-010-F	2044	149	58.34	38	GGTTTTTGTTAAGGGGGTTTT
MUPCDH-HRM-010-R	1896		59.6	48	TCCAATCCCTTCTACCCTATACC
MUPCDH-HRM-011-F	2156	172	59.76	39	TGTTATTTGGTAGGAGGGTTTGA
MUPCDH-HRM-011-R	1985		58.36	36	CCCCTCCTTCTAACAATAAACTAAA
MUPCDH-HRM-012-F	2252	119	59.64	35	ATATTGGGTTTGGGTTATGGTTT
MUPCDH-HRM-012-R	2134		59.76	39	TCAAACCCTCCTACCAAATAACA

Supplementary Table S2. PCR Primer sequences for EpiTYPER

Primer ID	sequence
MUPCDH-01-10F	aggaagagagGTTTTAGGGAGAGGTTATTGGGTAG
MUPCDH-01-T7R	cagtaatacgactcactatagggagaaggctATAAAAAAACCAAAAAAACTCAAAC
MUPCDH-02-10F	aggaagagagGGGGTTAATGAAAAGAGTAGGGTAA
MUPCDH-02-T7R	cagtaatacgactcactatagggagaaggctATCTCCCTCAAACAAAATTCCTAAA
MUPCDH-03-10F	aggaagagagAGGGGAGGAGTTAAGTTAGGGTAGT
MUPCDH-03-T7R	cagtaatacgactcactatagggagaaggctAAAATCAACCAAAAAACTCAAATCA
MUPCDH-04-10F	aggaagagagTTTTTTTGGTAGTGGGTTGGATT
MUPCDH-04-T7R	cagtaatacgactcactatagggagaaggctACACTAAACCTAAACCATAATCCCC
MUPCDH-05-10F	aggaagagagTTGTTGTGGTTTTTTTGTTGTTTA
MUPCDH-05-T7R	cagtaatacgactcactatagggagaaggctAAAAAACAACTAATTTCCCTAAATCC

Supplementary Fig. S1

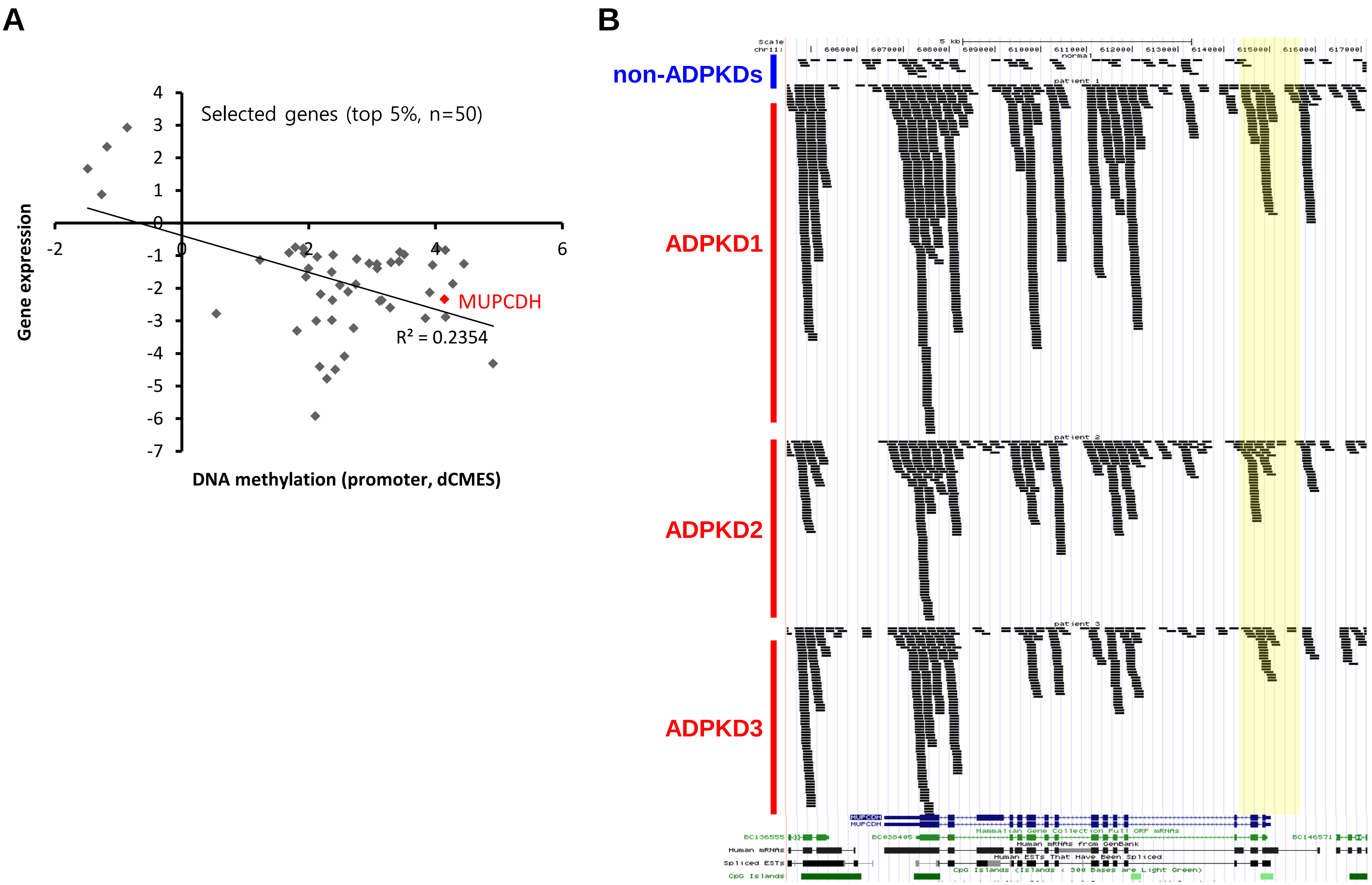

Supplementary Fig. S2

A

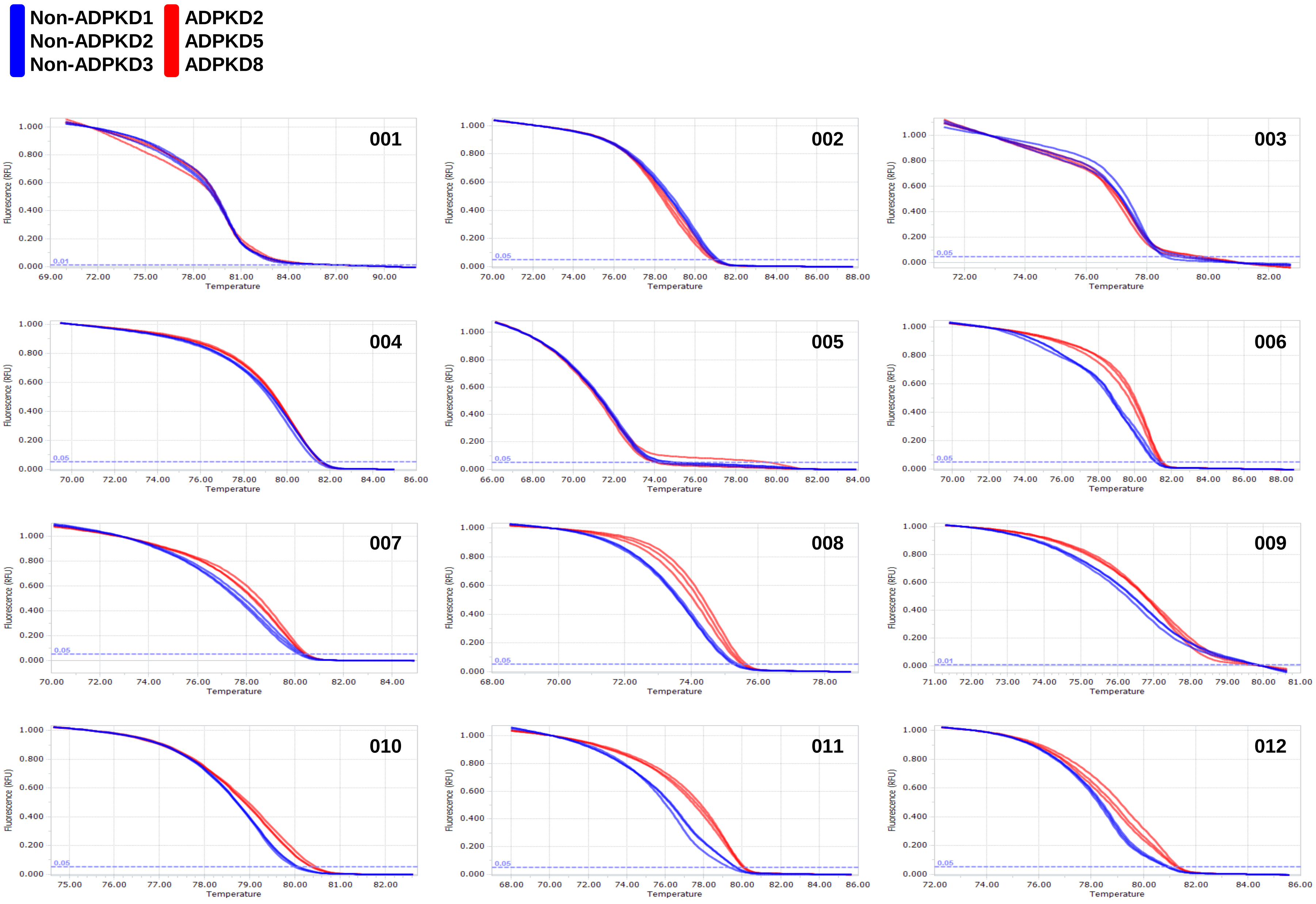

Supplementary Fig. S2

B

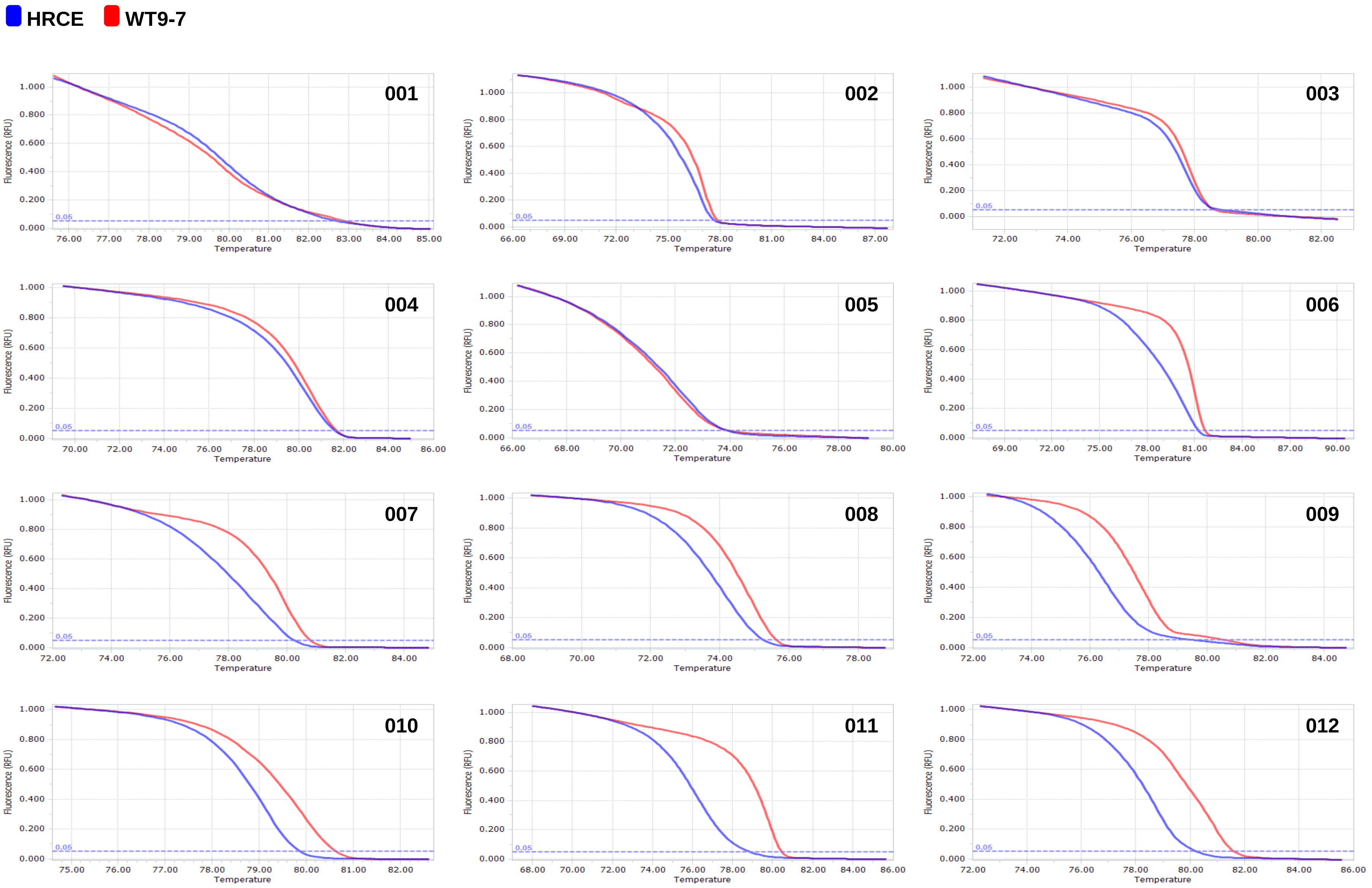

Supplementary Fig. S3

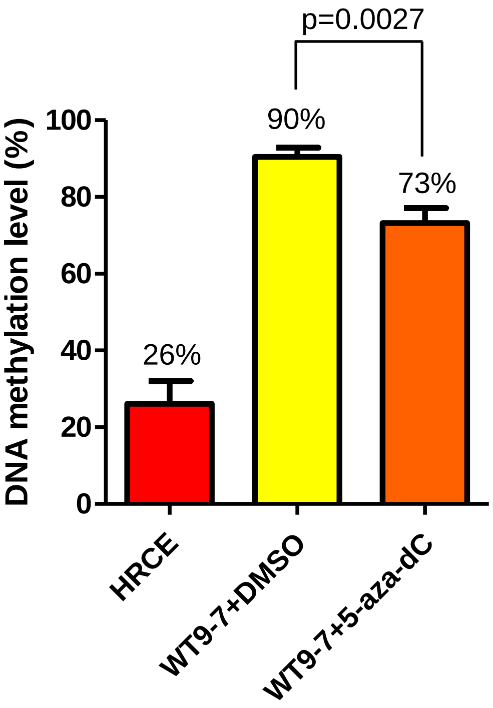

Supplementary Fig. S4

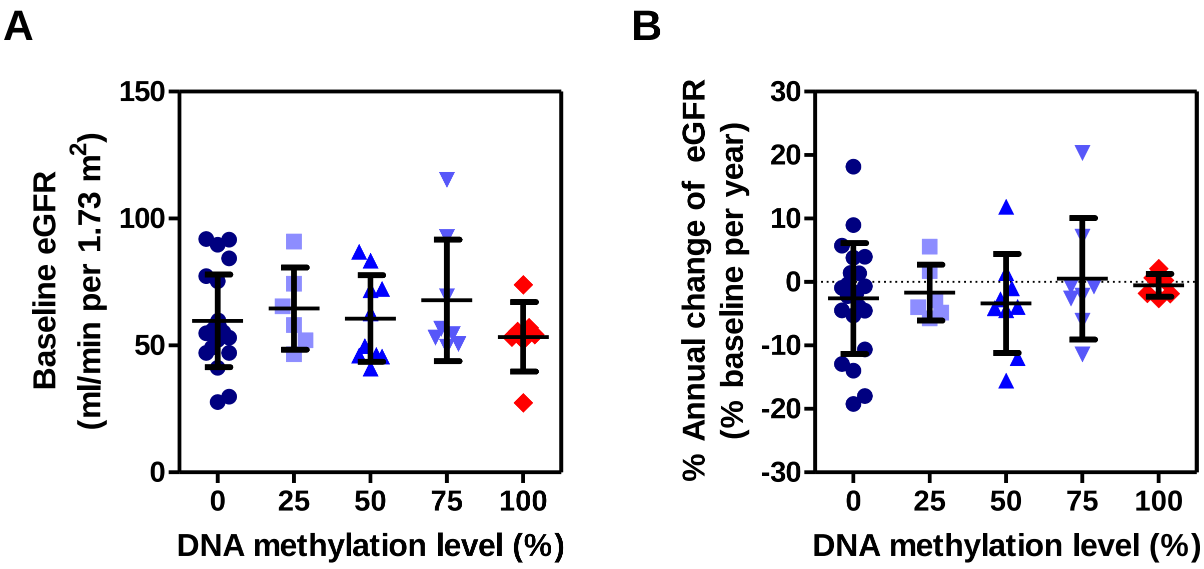

Supplementary Fig. S5

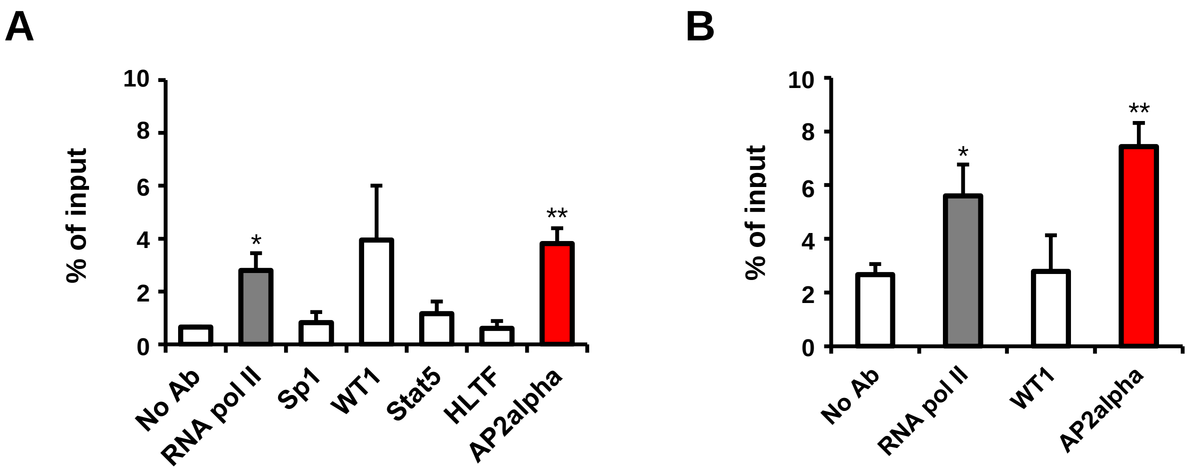

Supplementary Fig. S6

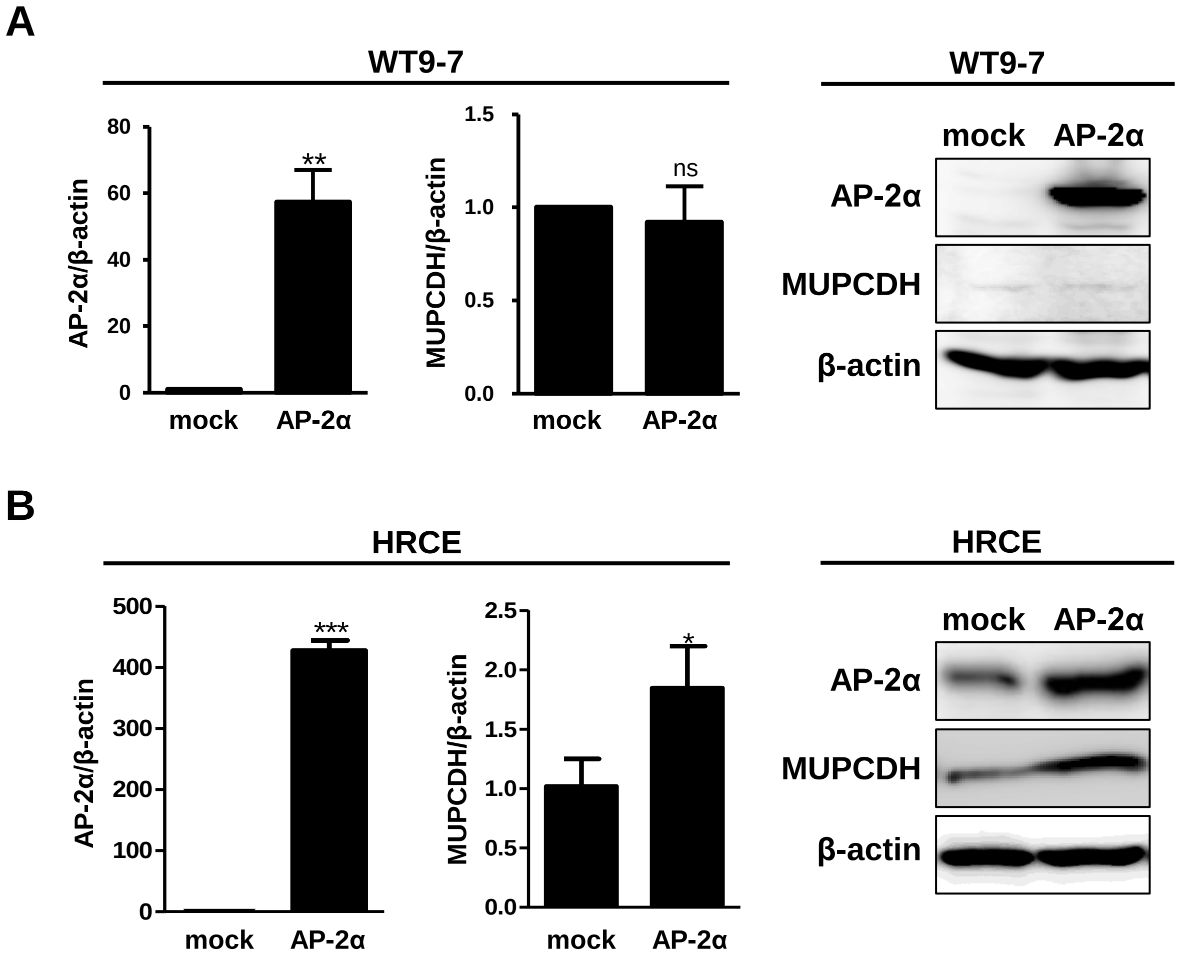

Supplement: Supplementary Information [file srep15238-s1.pdf]
